# Concomitant Targeting of Multiple Key Transcription Factors Effectively Disrupts Cancer Stem Cells Enriched in Side Population of Human Pancreatic Cancer Cells

**DOI:** 10.1371/journal.pone.0073942

**Published:** 2013-09-11

**Authors:** Xiyan Wang, Quentin Liu, Benxin Hou, Wei Zhang, Min Yan, Huimin Jia, Haijun Li, Dong Yan, Feimeng Zheng, Wei Ding, Chao Yi, Hai Wang

**Affiliations:** 1 Department of Hepatobiliary Surgery, Cancer Affiliated Hospital of Xinjiang Medical University, Urumqi, China; 2 State Key Laboratory of Oncology in South China, Sun Yat-sen University Cancer Center, Guangzhou, China; Univ of Bradford, United Kingdom

## Abstract

**Background:**

A major challenge in the treatment of pancreatic ductal adenocarcinoma is the failure of chemotherapy, which is likely due to the presence of the cancer stem cells (CSCs).

**Objective:**

To identify side population (SP) cells and characterize s-like properties in human pancreatic cancer cell lines (h-PCCLs) and to exploit the efficacy of concomitant targeting of multiple key transcription factors governing the stemness of pancreatic CSCs in suppressing CSC-like phenotypes.

**Methods:**

Flow cytometry and Hoechst 33342 DNA-binding dye efflux assay were used to sort SP and non-SP (NSP) cells from three h-PCCLs: PANC-1, SW1990, and BxPc-3. The self-renewal ability, invasiveness, migration and drug resistance of SP cells were evaluated. Expression of CSC marker genes was analyzed. Tumorigenicity was assessed using a xenograft model in nude mice. Effects of a complex decoy oligonucleotide (cdODN-SCO) designed to simultaneously targeting Sox2, Oct4 and c-Myc were assessed.

**Results:**

CSCs were enriched in the side proportion (SP) cells contained in the h-PCCLs and they possessed aggressive growth, invasion, migration and drug-resistance properties, compared with NSP cells. SP cells overexpressed stem cell markers CD133 and ALDH1, pluripotency maintaining factors Nanog, Sox2 and Oct4, oncogenic transcription factor c-Myc, signaling molecule Notch1, and drug resistant gene ABCG2. Moreover, SP cells consistently demonstrated significantly greater tumorigenicity than NSP cells in xenograft model of nude mice. CdODN–SOC efficiently suppressed all CSC properties and phenotypes, and minimized the tumorigenic capability of the SP cells and the resistance to chemotherapy. By comparison, the negative control failed to do so.

**Conclusion:**

The findings indicate that targeting the key genes conferring the stemness of CSCs can efficiently eliminate CSC-like phenotypes, and thus may be considered a new approach for cancer therapy. Specifically, the present study establishes the combination of Sox2/Oct4/c-Myc targeting as a potential anti-pancreatic cancer agent worthy of further studies in preclinical settings.

## Introduction

Pancreatic ductal adenocarcinoma (PDAC), known by its aggressiveness in nature, is a highly lethal malignancy that is usually diagnosed at a late stage for which optimal therapeutic options have been skipped [1]. The poor prognosis may be explained by the late detection of the neoplastic process, lack of effective treatment, and limited knowledge of its biological characteristics. Hence, better understanding of the cellular/molecular properties associated with this condition is urgently needed to explore novel venues of diagnostics and treatment of this dismal disease.

Emerging evidence suggests that malignant tumors are composed of a small subset of distinct cancer cells, termed "cancer stem cells" (CSCs), typically less than 5% of total cancer cells based on cell surface marker expression [2–6]. CSCs are found in a sub-population of cells that is distinct from the main population within tumors or hematological cancers, called “side population” cells (SP cells) exhibiting stem cell-like characteristics. CSCs possess the capacity to self-renew and to generate the heterogeneous lineages of cancer cells that comprise the tumor; they are therefore tumorigenic, in contrast to other non-tumorigenic cancer cells, and are essential drivers for tumor progression and metastasis. Clinically even more important, however, is the fact that CSCs also confer virulence via immune system evasion and multidrug resistance to chemotherapy and radiotherapy resulting in their relative enrichment during treatment and rapid relapse of disease [2–6]. The efficacy of cancer treatments is often measured by the ablation fraction of tumor mass, and conventional chemotherapies kill differentiated or differentiating cells, which form the bulk of the tumor but are unable to generate new cells. As CSCs form a rather small proportion of the tumor, they could remain un-attacked, causing a relapse of the disease. Therefore, development of specific therapies targeted at CSCs holds tremendous hope for improvement of survival and quality of life of cancer patients, especially for sufferers of metastatic disease.

CSCs have been identified in PDAC and pancreatic cancer cell lines by several laboratories [7–14]. Human pancreatic CSCs expressing high levels of CD133, CD24, CD44, ESA, and aldehyde dehydrogenase (ALDH1) also have more abundant Nanog, Oct4, Notch1, MDR1 and ABCG2 than normal pancreatic tissues and primary pancreatic cancer cells [10–12,14,15]. It appears that PDAC does not only contain one homogeneous population of CSCs rather than diverse subpopulations that may have evolved during tumor progression, based on the use of combinations of surface markers that allow their isolation, propagation, and further characterization. One of these populations is called migrating CSCs and these cells are capable of evading the primary tumor and traveling to distant sites such as the liver as the preferred site of metastatic spread.

Therefore, successful treatments of cancers not only rely on identifying the source of cancer cells and anticancer therapy for the differentiated cancer cells, but also on finding potential CSC population and reaching sufficient destroying of CSCs in the tumor. Indeed, CSC-targeted approaches have demonstrated great promise in preclinical models [2-6]. These approaches include direct strategies, such as ablation by targeting molecular markers of CSCs or CSC-specific pathways, reversal of resistance mechanisms, and differentiation therapy, and indirect strategies, such as antiangiogenic therapy, immunotherapeutic approaches, and disruption of protumorigenic interactions between CSCs and their microenvironment.

The present study was conducted to achieve the following two primary goals. First, to have a comprehensive characterization of the CSC populations in human pancreatic cancer cell lines, we compared the SP cells in BxPc-3, PANC-1 and SW1990 cell lines. Second, to exploit the possibility of simultaneously targeting multiple transcription factors governing the stemness of pancreatic CSCs to disrupt CSCs thereby tumorigenesis and metastasis, we designed a decoy oligonucleotides fragment by the “one agent, multiple targets” concept and tested the efficacy of this molecule to silence the stemness of pancreatic CSCs.

## Materials and Methods

### Ethics statement

The used of all animals in this study was approved by, and all operative procedures and animal care strictly conformed to Guidelines set by, the Animal Ethics Committee of the Xinjiang Medical University and the Sun Yat-sen University.

### Cell culture

The human pancreatic cancer cell lines BxPc-3, PANC-1, and SW1990 originally established from human primary pancreatic adenocarcinoma by ATCC were purchased from Shanghai Cell Bank (Shanghai, China) and propagated in our laboratory. All cell lines were maintained in Dulbecco’s modified eagle medium (DMEM). Medium was supplemented with 1% penicillin/streptomycin, 3 mM L-glutamine, and 10% fetal bovine serum (FBS) (Gibco). Cells were maintained in a humidified incubator at 37°C containing 5% CO_2_.

### Flow cytometry analysis and cell sorting

SP cells are a small group of cells that stain faintly or not at all when treated with Hoechst 33342 dye. These cells can be isolated using flow cytometry protocols initially established by Goodell et al. in a study of murine bone marrow [16,17]. H-PCCLs were detached from the culture dish with 0.25% trypsin, washed with phosphate-buffered saline (PBS), and suspended at 1×10^6^ cells/ml in culture medium containing 2% FBS. The tumor cells and cell lines were incubated with Hoechst 33342 dye (Sigma-Aldrich) at a final concentration of 5 µg/ml with or without verapamil (100 µM; Sigma-Aldrich) at 37°C for 90 min with intermittent shaking every 15 min. Verapamil was used to verify the SP phenotype, as it can reduce the side branch by blocking the multidrug transporters. After washed twice with PBS, the cells were resuspended in ice-cold PBS containing 2% FBS, passed through a 40-µm mesh filter to obtain single-cell suspensions, and kept on ice until flow cytometry analysis. Propidium iodide (PI; 2 μg/ml; Sigma-Aldrich) was added to label and exclude dead cells. Cell analysis and fluorescence-activated cell sorting (FACS) were conducted using a FACS Vantage SE equipped with the version 6.0 FACS DIVA software (BD Biosciences, Erembodegem, Belgium). Hoechst 33342 dye was excited by a 350-nm ultraviolet laser, and fluorescence emission was dual-wavelength analyzed (Hoechst blue with 402-450 nm filter; Hoechst red with 650-670 nm filter).

### Cell proliferation and cell cycle assays

One thousand sorted SP and non-SP (NSP) cells were seeded into separate well of a 96-well plate in triplicate and cultured in Leibovitz’s L-15 medium with 1% penicillin/streptomycin and 10% FBS for 3 days. Growth was measured using the 3-(4,5-dimethylthiazol-2-yl)-2,5-diphenyltetrazolium bromide (MTT) method. Briefly, 20 µl MTT solution (5 mg/ml in PBS; Sigma) was added to each well and incubated at 37°C for 4 h. A 150-µl aliquot of DMSO was then added, and absorbance was measured by a Microplate Reader (Multiscan MK3, Thermo Labsystem, USA) at a wavelength of 490 nm.

For cell-cycle assay, 5×10^5^ freshly sorted cells were washed twice with PBS and fixed with 2 ml of 70% ice-cold ethanol at 4°C overnight. The cells were then transferred to PBS, stained with 20 µg/ml PI and 1 mg/ml RNase, and analyzed using flow cytometry. The results are expressed as the percentage of cells in each phase of the cell cycle.

### Clone formation assays

The following steps were applied for the assays(1). Agar (10 g/l; DNA grade) was melted in the microwave and 2× DMEM supplemented with 200 ml/l FBS was warmed to 40^o^C in a water bath. Equal volumes of these two solutions were then mixed to yield a new solution of 5 g/l agar + 1× DMEM + 100 ml/l FBS(2). Next, 1 ml of mixed solution was added to each well of a 6-well plate to form the base agar(3). Then, 7 g/l agar (DNA grade) was melted in the microwave and cooled to 40^o^C in a water bath, and 2× DMEM and 200 ml/l FBS were warmed to the same temperature(4). Freshly sorted SP and non-SP cells were passed through a 40-µm filter to provide a single cell suspension and were counted. Three ml DMEM, 1.5 ml 2× DMEM containing 200 ml/l FBS and 1.5 ml agar including 500 sorted cells were then mixed together, and a 1.5 ml cell suspension of this solution was placed into each well of a 6-well plate as the top agar(5). Finally, 100 cells were seeded into each well, and were incubated at 37^o^C in a humidified incubator for 2-3 wk. Colonies were either left unstained, or were stained with 5 g/l MTT (Sigma-Aldrich) for 1 h, and counted under a dissecting microscope (the procedure was repeated three times)(6). After SP cells were seeded in soft agar assays, colonies containing more than 50 cells (primary colony) were removed from soft agar with sterile Pasteur pipettes, treated with trypsin and mechanically dissociated into single cells. Then, step (5) was repeated for the secondary colony.

### Sphere formation assays

Sorted SP and NSP cells from the three h-PCCLs were passed through a 40-µm filter to obtain a single cell suspension. Then, 4 ml medium containing 100 cells were added to 6-well plates with serum-free culture medium DMEM-F12 (Invitrogen-Life Technologies), supplemented with epidermal growth factor (10 µg/l), insulin (20 mg/L) and bFGF (10 µg/l). Aliquots of epidermal growth factor, insulin and basic fibroblast growth factor were added twice a week. After 10 d, plates were visually assayed for the formation of floating spheres and spheres were counted under a dissecting microscope.

### Invasion assay

Invasiveness of sorted SP and NSP cells was determined using 6-well Matrigel invasion chambers (BD Biosciences Discovery Labware). Cells were seeded in the top chamber onto the Matrigel coated Membrane (24-well insert; pore size, 8 mm; Corning Costar) at 2×10^5^ per insert in serum-free DMEM at 37^o^C with 5% CO_2_ for 48 h. Outer wells were filled with DMEM containing 5% FBS as chemo-attractant. The non-invading cells were removed by swabbing top layer of Matrigel with Q-tip. The membrane containing invading cells was stained with hematoxylin for 3 min, and then washed and mounted onto slides. The invading cells on the entire membrane were counted under a light microscope at 40× objective.

### Transwell migration assay

Sorted SP or NSP cells at 1×10^5^ were plated in the top chamber onto the non-coated membrane (24-well insert; pore size, 8 mm; Corning Costar) and allowed to migrate toward serum-containing medium in the lower chamber. Cells were fixed after 24 h of incubation with methanol and stained with 0.1% crystal violet (2 mg/ml, Sigma-Aldrich). The number of cells invading through the membrane was counted under a light microscope (40×, three random fields per well).

### Drug resistance analysis

Aliquots of 2×10^3^ freshly sorted SP and NSP cells were seeded in 96-well plates in triplicate with 200 µl DMEM per well. After a 12-h recovery period, cells were exposed to various concentrations of gemcitabine for 72 h. The effects on cell growth were examined by the MTT assay as described above. The cell survival rate (SR) was calculated using the formula: SR = (mean absorbance of the test well / mean absorbance of the control) × 100%; the cell resistance rate (RR) was calculated using the formula: RR =100% -SR.

Apoptotic cells were determined by fluorescein isothiocyanate (FITC)-Annexin-V methods. In brief, freshly sorted cells were seeded into 6-well plates at a density of 1×10^5^ cells/well. After 12 h of recovery, the cells were exposed to varying concentrations of gemcitabine for 48 h. The cells were then stained with Annexin-V and PI using the ApoAlert™ Annexin V Apoptosis Kit (Clontech, Cosete Tech, Jinan) as per the manufacturer’s protocol. Briefly, cells were harvested by trypsinization and washed twice with cold PBS. The pellets were resuspended in 100 µl 1× Annexin binding buffer and 5 µl FITC-Annexin-V. A 1-µl working solution of PI at 100 µg/ml was added to each 100 µl of cell suspension. The cells were incubated on ice for 1 h, washed again with cold PBS and resuspended in 300 µl 1× Annexin-binding buffer. The stained cells were immediately analyzed by flow cytometry.

### In vitro differentiation study

Freshly sorted SP and NSP cells were cultured at a density of 1×10^5^ cells/well in a 6-well culture plate in Leibovitz’s L-15 medium with 1% penicillin/streptomycin and 10% FBS and incubated at 37°C with 5% CO_2_. After 7 days, SP- and non-SP derived cells were re-analyzed for the presence of an SP fraction using the methods described above.

### Tumorigenicity assay in vivo

Athymic 4- to 5-week-old mice (BALB/c nude mice) were supplied by Slac Laboratory Animal (Shanghai). Mice were housed and maintained in laminar flow cabinets under pathogen-free conditions. Groups of mice were orthotopically inoculated s.c. to the left dorsal flank of mice with freshly sorted SP cells at 1×10^3^, 1×10^4^, 1×10^5^, and 1×10^6^ or NSP cells at 1×10^3^, 1×10^4^, 1×10^5^, and 1×10^6^ (four mice per group). Tumor growth was monitored every 2 days after second week of inoculation. The mice were sacrificed at day 50 or when the tumors grow to a maximum of 1,000 mm^3^. Tumor volume was calculated by the formula 0.52 × length × width^2^. Fold difference in tumorigenicity was calculated by the following formula: NSP_min_ / SP_min_, where NSP_min_ is the minimum number of NSP cells needed to generate a tumor and SP_min_ is the minimum number of SP cells needed to generate a tumor. Finally, tumors were also digested to make single-cell suspension for SP re-analysis by the Hoechst33342 dye efflux assay as described above.

When the h-PCCL-induced tumors reached a volume of approximately 200 mm^3^, one group of mice received gemcitabine (200 mg/kg body weight i.p., 1 injection every 3 days, 6 injections in total) and the other group (bearing the corresponding tumors) was injected with vehicle (0.9% NaCl; control group). Tumor diameter was measured every 3 days after the first injection. Gemcitabine was considered effective when tumor volume decreased at least 50%. Three days after the last injection, mice were euthanized and tumors analyzed to determine the proportion of SP cells as described above.

### RNA extraction and real-time PCR analysis

Sorted cells and cultured cells were treated with TRIzol reagent (Invitrogen China Limited, Shanghai) and mixed thoroughly with pipetting. The Trizol-lysates were then mixed with chloroform and centrifuged at 15,000× g for 15 min. Following centrifugation, total RNA were obtained by isopropanol precipitation according to the manufacturer’s instructions.

Single strand cDNA was reverse transcribed from total RNA using random primer under standard conditions with the High Capacity cDNA reverse transcription kit (Applied Biosystems, Foster City, CA, USA). Quantitative real-time PCR with total cDNA was performed with SYBR Green Master Mix Real-Time Core Reagents on an ABI 7500 (Applied Biosystems) according to the manufacturer’s instructions. The amplifications were carried out at 95°C for 10 sec followed by 40 cycles of 95°C for 5 s, 60°C for 30 s. To quantify the relative expression of each gene, the Ct (threshold cycle) values were normalized for endogenous reference ( ^Δ^Ct = Ct target- Ct β-actin) and compared with a calibrator, using the " ^∆∆^Ct method" ( ^∆∆^Ct = ^Δ^Ct_sample_ - ^Δ^Ct_calibrator_). Using the ^∆∆^Ct value, relative expression was calculated (^2-∆∆^Ct). As the ^Δ^Ct method is only applicable when the amplification efficiencies of the target and the reference are essentially equal, we determined the efficiencies for 5 dilutions, and the delta Ct values (Ct_target_-Ct_β-actin_) were plotted against the dilution (log). The slope of the fitted line was then determined. All samples were tested in triplicate, and the average values were used for quantification.

### Western blot analysis

Membrane and cytosolic protein samples were extracted from SP and NSP cells from all three h-PCCLs. Protein concentration was determined by the protein content was determined by BCA Protein Assay Kit using bovine serum albumin as the standard (Pierce, USA). Equal amounts of protein samples (~50 µg) were fractionated by SDS-PAGE (12% polyacrylamide gels) and electrophoretically transferred to PVDF membrane (Millipore, Bedford, MA) using a Mini Trans-blot (Bio-Rad Laboratories, Shanghai). The protein samples (membrane sample for ABCG2 and CD133; cytosolic sample for ALDH1, Oct4, Sox2 and Nanog) were incubated with the primary antibodies in 1:200 at 4°C overnight. Affinity purified rabbit polyclonal anti-ABCG2 (Shanghai Ruiqi Biological Technology Co. LTD), rabbit polyclonal anti-CD133 (Shanghai XiangSheng Biotech Co. LTD), rabbit polyclonal anti-Oct4 (Cell Signaling Shanghai), rabbit polyclonal anti-Sox2 (Shanghai ExCell Bio Co. LTD), rabbit polyclonal anti-ALDH1 (Santa Cruz Biotechnology Inc., CA), and rabbit polyclonal anti-Nanog (Santa Cruz Biotechnology Inc., CA) were used as the primary antibodies. Next day, the membrane was incubated with secondary antibodies (Molecular Probes) diluted in PBS for 2 h at room temperature. Finally, the membrane was rinsed with PBS before scanning using the Infrared Imaging System (LI-COR Biosciences). GAPDH was used as an internal control for equal input of protein samples, using anti-GAPDH antibody. Western blot bands were quantified using QuantityOne software by measuring the band intensity (Area × OD) for each group and normalizing to GAPDH. The final results are expressed as fold changes by normalizing the data to the control values.

### Preparation of complex decoy oligodeoxynucleotide (cdODN)

We designed a cdODN bearing consensus sequences for four transcription factors Sox2, Oct4, and c-Myc, following the principle established by Gao et al. [18]. Single-stranded phosphorothioate oligodeoxynucleotides were synthesized by IDT incorporation (Coralville, IA). The ODNs were washed in 70% ethanol, dried and dissolved in sterilized Tris-EDTA buffer (10 mM Tris + 1 mM EDTA). The supernatant was purified using Micro Bio-spin30 columns (BioRad, Shanghai) and quantified by spectrophotometry. The double-stranded dODNs were then prepared by annealing complementary single stranded oligodeoxynucleotides by heating to 95^o^C for 10 min followed by cooling to room temperature (RT) slowly over 2 h. For convenience, we designated this cdODN-SOC. A negative control fragment (NC-cdODN) carrying base pair replacement was also synthesized.

### Electrophoretic mobility shift assay (EMSA)

EMSA was conducted according the procedures described in Gao et al. [18]. Briefly, cdODN-SOC or NC–ODN was labeled with [γ-^32^P] ATP. The sample was then loaded into the G-25 column and centrifuged at 7000*g* for 2 min. The recombinant human proteins Sox2, Oct4 and c-Myc were (Santa Cruz Biotechnology) were incubated separately cdODN-SOC or NC–ODN at RT for 15 min in 10 µl of H_2_O and 8 µl of master mix (12×) containing 1 M Tris-HCl, pH 7.5, 0.5 M EDTA, 5 M NaCl, 1 M dithiothreitol, 50% glycerol, 100 µg/µl bovine serum albumin, and 1 µg/µl poly(dIdC). For supershift experiments, antibodies (1 µg) were included in the reaction. For competition experiments, unlabeled cdODN-SOC in 100-fold excess of the labeled dODNs was added in the binding reactions. DNA-protein complexes were separated by nondenaturing polyacrylamide gel (7.5% in 0.4 × Tris-borate/EDTA) electrophoresis. Gels were dried and filmed.

### Transfection of cdODN-SOC into cell lines

The sorted SP or NSP cells were transfected with cdODN-SOC using Lipofectamine 2000 (Invitrogen China Limited, Shanghai). The cells were seeded in 96-well tissue culture plates. At 50% confluence, the cells were washed with serum-free medium once and then incubated with 50 µl fresh fetal bovine serum (FBS)-free medium. Decoy ODNs of varying concentrations and lipofectamine (0.25 µl) were separately mixed with 25 µl of Opti-MEM® I Reduced Serum Medium (Invitrogen China Limited, Shanghai) for 5 min. Then the two mixtures were combined and incubated for 20 min at RT. The lipofectamine: cdODN mixture was added dropwise to the cells and incubated at 37°C for 5 h. Subsequently, 25 µl fresh medium containing 30% FBS was added to the well and the cells were maintained in the culture until use.

### Administration of cdODN-SOC to BALB/c nude mice

When tumor size reached ~200 mm^3^ (approximately 7 days after inoculation), cdODN-SOC was administered daily by a single intratumoral injection (20 µl of 100 nM cdODN-SOC mixed with Lipofectamine 2000). Tumor growth was monitored regularly, and the volume (V) of tumors at day 5 after cdODN-SOC treatment was calculated using the formula V = 1/2 × length × (width)^2^.

### Data analysis

Data are presented as mean ± SEM for all experimental data. Group comparisons were performed using one-way ANOVA. Post hoc analyses of significant main effects were further examined using Fisher’s PLSD tests. A two-tailed *P*<0.05 value was taken to indicate a statistically significant difference between two groups. Statistical analyses were performed with SPSS 11.5 and curve-fitting was performed in Graphpad Prism software.

## Results

### Characteristics of human pancreatic cancer cell lines in culture

**Figure 1:**
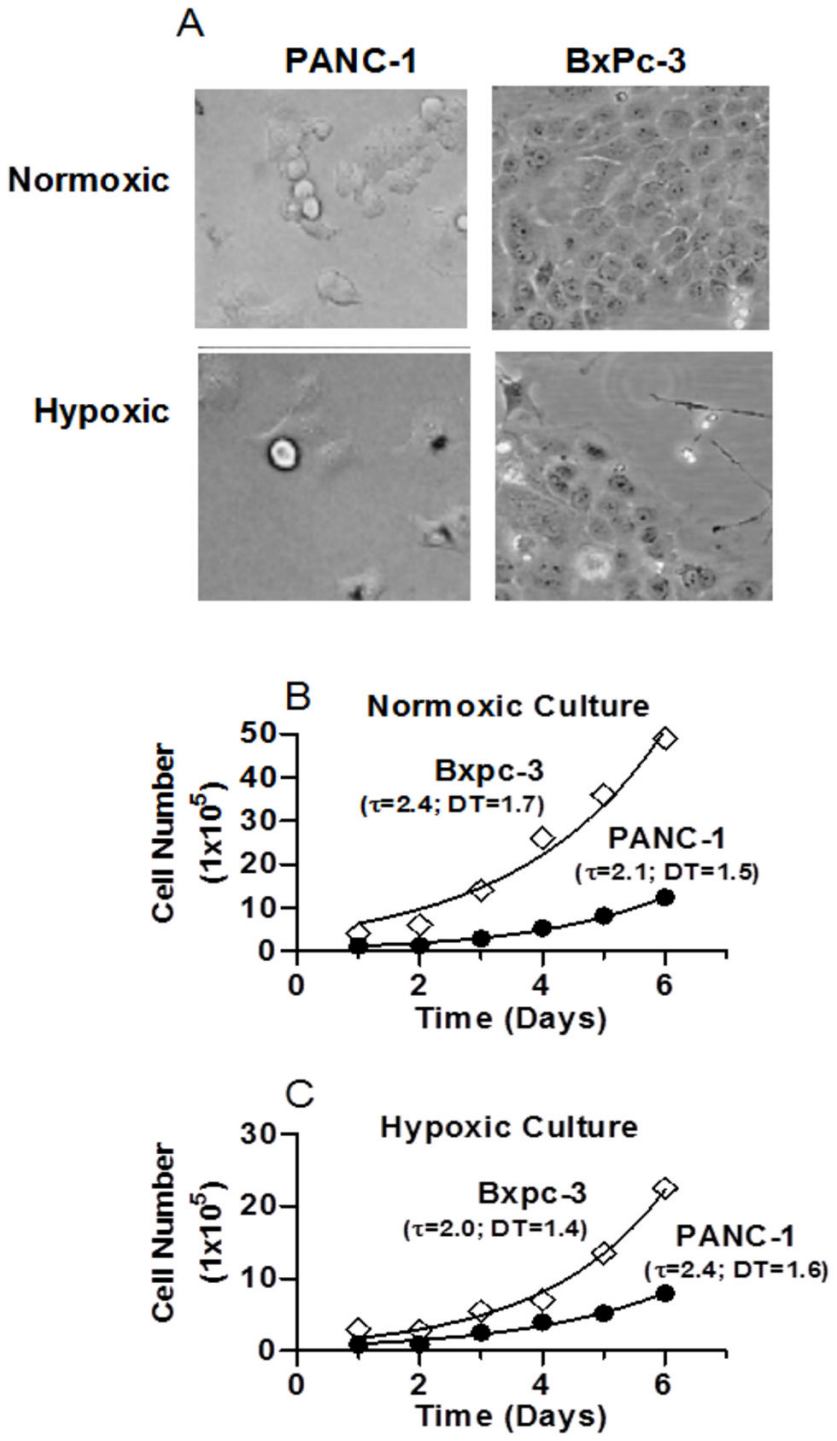
Growth characteristics of human pancreatic cancer cell lines in culture. (**A**) Representative photomicrographs of PANC-1 and BxPc-3 cells 72 h after seeding. (**B**) & (**C**) Growth curves of PANC-1 and BxPc-3 cells. Symbols are experimental data and curves represent the best fits to exponential growth equation: Y = Y0 × exp(k × X), where Y0 is the Y value when X (time) is zero and K is the rate constant. τ is the time constant (day) and DT (doubling-time) is in the time units of the X axis, computed as ln_2_/K.

In normal oxygen culture medium, the human pancreatic cancer cell lines (h-PCCLs) PANC-1, SW1990, and BxPc-3 all demonstrated clear cell boundaries, rich cytoplasm, distinct nucleus, and visible nucleoli, many ongoing dividing cells with even cell morphology mainly in the epithelial-like polygonal shape and rarely in the spindle shape under an inverted microscope (Figure **1A**). Under hypoxic culturing conditions, however, cell boundaries became hazy and cell size became larger but with shrunk cytoplasm, enlarged nucleus, and fuzzy nucleolus with less dividing cells and more spindle-shaped cells.

Growth of PANC-1 and BxPc-3 cells was assessed. With initial equal seeding density, these cells showed similar rates of growth under normoxic conditions. Three days after seeding, the cells entered the logarithmic growth phase and formed a growth zone which was gradually expanding radially. The cells had uniform morphology and orderly aligned (Figure **1B**). By comparison, under hypoxic culturing conditions, the cell growth was substantially slowed with no apparent logarithmic growth phase (Figure **1C**). Cells were disarranged and cells within the growth zone showed heterogeneous morphology.

### Identification of SP cells from human pancreatic cancer cell lines and effects of cdODN-SOC

**Figure 2:**
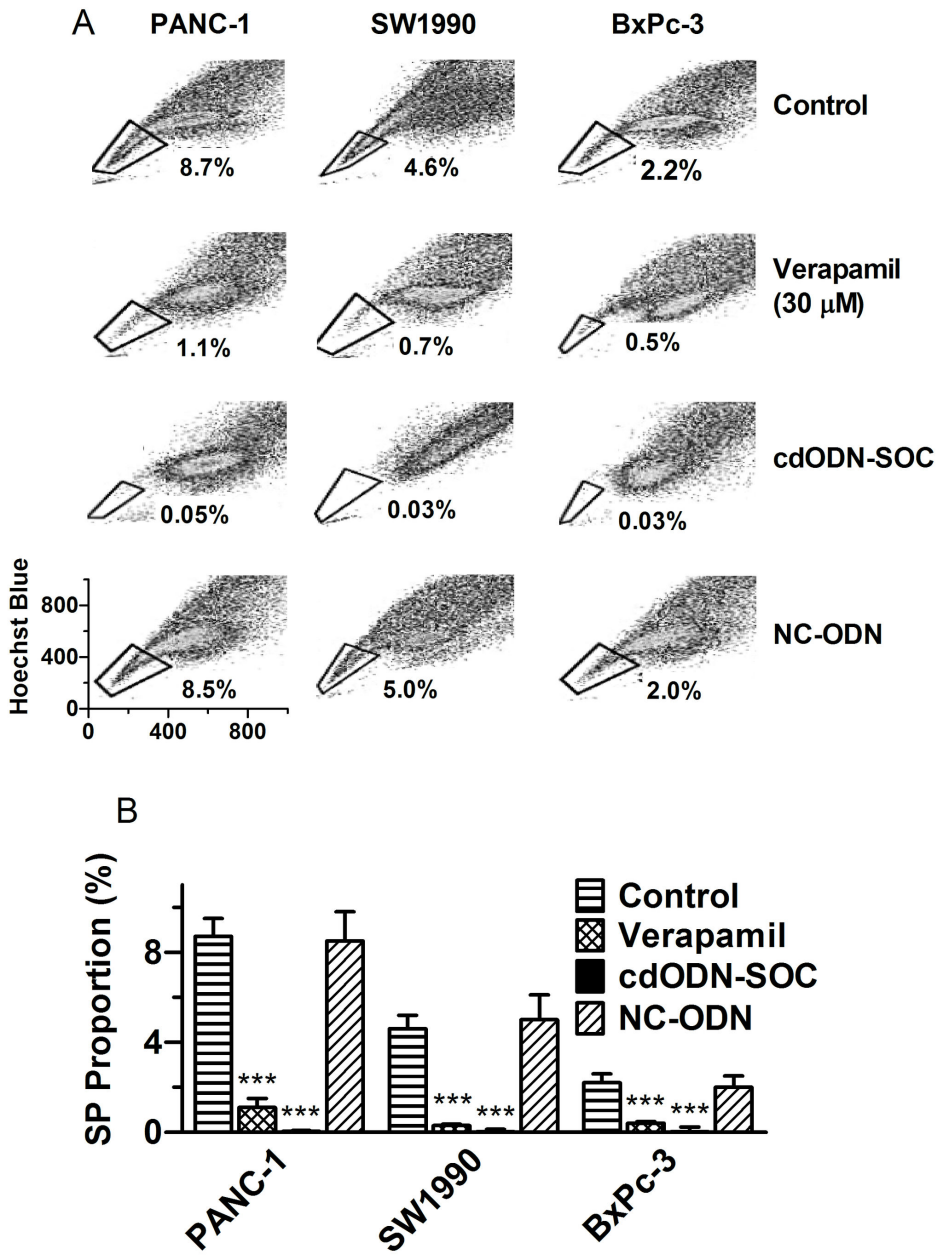
Analysis of the side population (SP) in three human pancreatic cancer cell lines PANC-1, SW1990, and BxPc-3, using flow cytometry and Hoechst33342 dye. (**A**) Examples of floe cytometry analysis of SP cells in the presence or absence of 30 µM verapamil, cdODN-SOC (a complex decoy oligodeoxynucleotide carrying *cis*-elements for Sox2, Oct4 and c-Myc), or NC–ODN (negative control cdODN). (**B**) The SP proportions as percentage of total population. ***p<0.001 *vs* Control; n=4.

According to our Hoechst33342‒FACS analysis, the h-PCCLs PANC-1, SW1990, and BxPc-3 all contained a small subpopulation of SP cells with proportions of 8.7±0.8, 4.6±0.6, and 2.2±0.4%, respectively (Figure **2A,B**). The SP fraction in cell lines was substantially diminished in the presence of verapamil.

To exploit whether the SP cells could be converted to NSP by targeting the key molecules determining the stemness of CSCs, we tested the effects of a complex decoy oligodeoxynucleotides fragment (cdODN) on SP cells. The complex decoy oligodeoxynucleotide (cdODN-SOC) designed for this study contained the typical *cis*-acting elements for Sox2, Oct4, and c-Myc. Strikingly, after pretreated with cdODN-SOC for 48 h, SP cells were nearly vanished in all three h-PCCLs, with 0.05±0.03, 0.03±0.1 and 0.03±0.2% for PANC-1, SW1990, and BxPc-3, respectively (Figure **2A,B**). As a negative control, NC-cdODN did not significantly alter the SP fractions in these cell lines.

**Figure 3:**
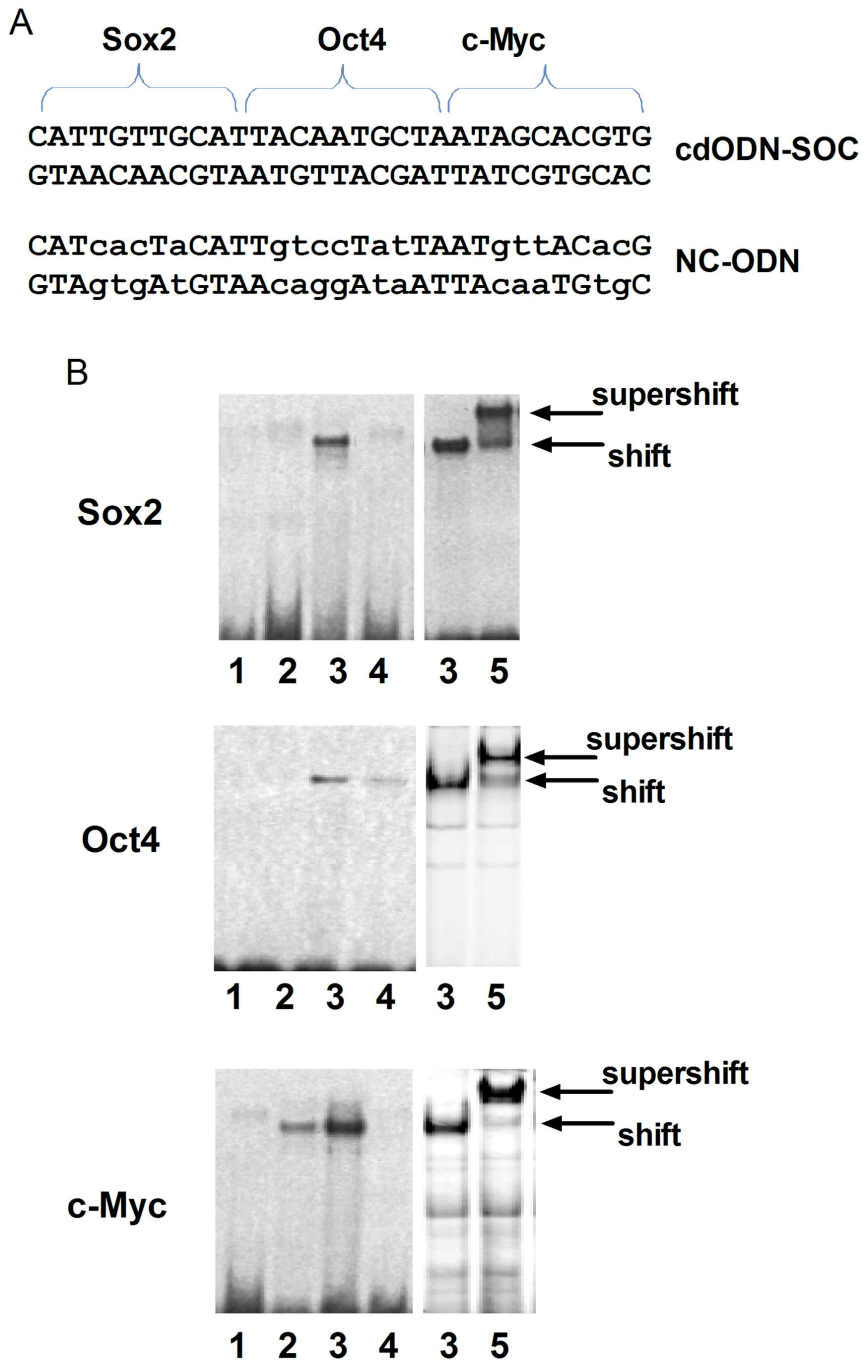
Validation of the ability of cdODN-SOC to bind target transcription factors, assessed by electrophoretic mobility shift assay (EMSA). (**A**) The sequences of the cdODN-SOC carrying *cis*-elements for Sox2, Oct4 and c-Myc and the negative control ODN with nucleotide replacements (NC–ODN). (**B**) Examples of EMSA images showing the ability of cdODN-SOC to bind human recombinant Sox2, Oct4 or c-Myc protein. The shift bands indicate the positions of the DNA-protein complexes and the supershift bands indicate the specific DNA-protein binding picked up by the respective antibody. Lane labels: 1, control with no proteins; 2: in the presence of cold or unlabeled cdODN-SOC; 3: in the presence of cdODN-SOC; 4: in the presence of NC–ODN; and 5: with antibody.

The sequence of this cdODN-SOC is shown in Figure **3A** and the ability of this cd-ODN-SOC to bind Sox2, Oct4 and c-Myc proteins was verified by our EMSA in conjunction with supershift using antibodies directed against these transcription factors. As depicted in Figure **3B**, the shifted bands indicate cdODN-protein binding and the supersfift bands indicated the specific cdODN-protein binding.

### Differential gene expression between SP and NSP cells

**Figure 4:**
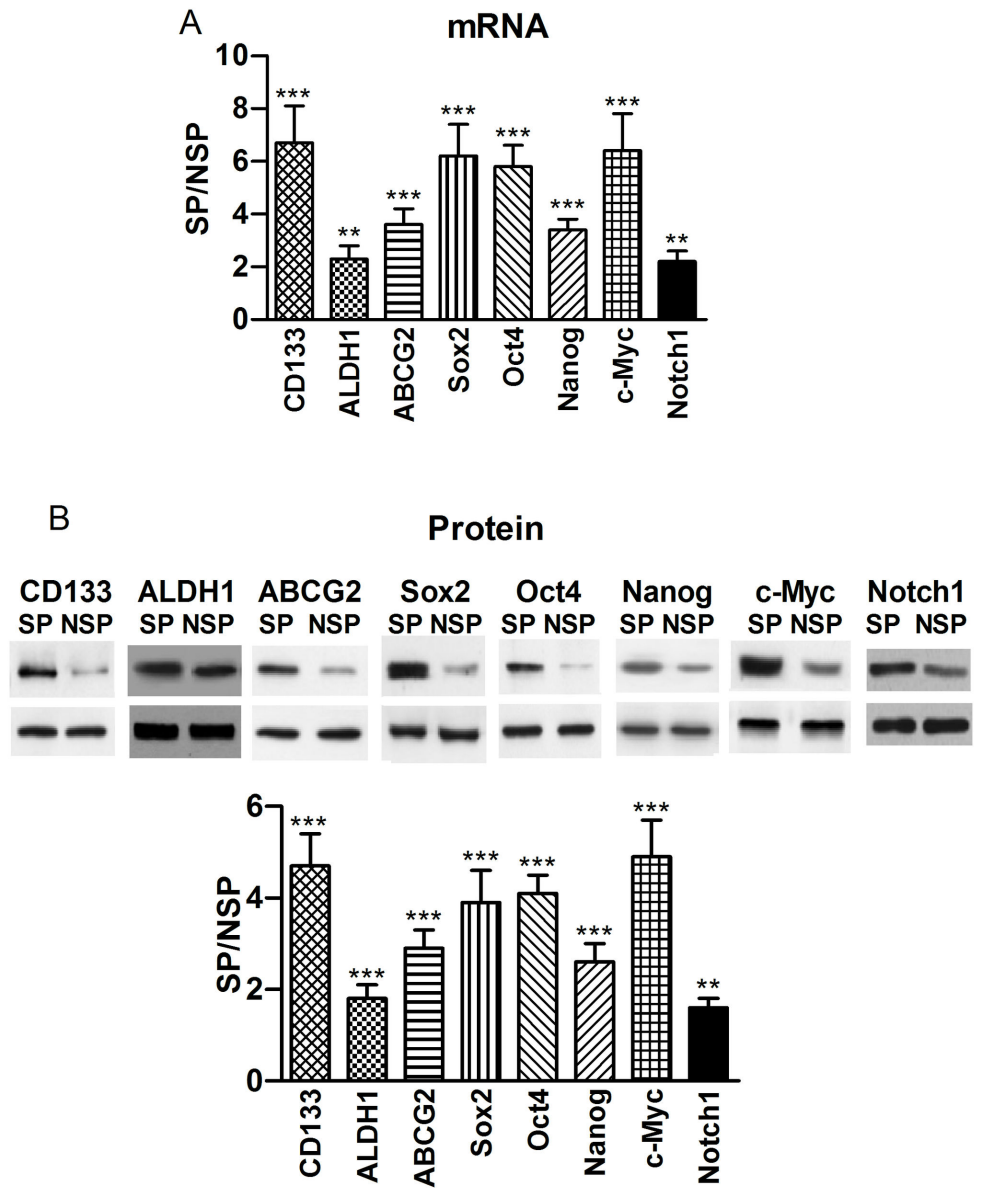
Differential gene expression between PANC-1 SP and NSP cells. (**A**) Expression of CD133, ALDH1, ABCG2, Sox2, Oct4, Nanog, c-Myc, and Notch1 at the mRNA level, determined by real-time RT-PCR. The values were obtained by first normalized to GAPDH for internal control and then presented as a ratio of SP over NSP. **p<0.01 SP *vs* NSP; ***p<0.001 SP *vs* NSP; n=4. (**B**) Expression of CD133, ALDH1, ABCG2, Sox2, Oct4, Nanog, c-Myc, and Notch1 at the protein level, determined by Western blot analysis. The values were obtained by first normalized to GAPDH for internal control and then presented as a ratio of SP over NSP. CD133: 120 kDa; ALDH1: 55 kDa; ABCG2: 72 kDa; Sox2: 40 kDa; Oct4: 45 kDa; Nanog: 35 kDa; c-Myc: 62 kDa; Notch1: 300 kDa. **p<0.01 SP *vs* NSP; ***p<0.001 SP *vs* NSP; n=4 (Similar results were observed in SW1990 and BxPc-3 cells; Figure S1 and Figure S2 online).

We next compared the expression of stem cell markers CD133 and ALDH1 (aldehyde dehydrogenase-1) [19,20], pluripotency maintaining factors Nanog, Sox2 and Oct4, oncogenic transcription factor c-Myc, signaling molecule Notch1, and drug resistant gene ABCG2 [21,22] in sorted SP and non-SP (NSP) cells by qRT-PCR for the transcript level and Western blot at the protein level. As illustrated in Figure **4**, SP cells from PANC-1 expressed substantially more abundant transcripts of these genes compared to NSP cells, clearly indicating the stem-cell characteristics of SP cells. The results from Western blot analysis were consistent with the changes of mRNA levels (Figure **4B**). SP cells from other two h-PCCLs showed quantitatively the same results (Figure **S1** and Figure **S2** online).

### CdODN–SOC suppresses growth, invasion and migration of human pancreatic CSCs

These above results suggested that SP cells isolated from h-PCCLs harbor CSC-like properties that may be related to the aggressiveness of pancreatic adenocarcinoma. To test whether the adverse CSC-like properties could be eliminated by targeting the key genes that confer the CSC-like properties of SP cells, we followed the “one agent, multiple targets” concept [18] and tested the effects of cdODN-SOC on the properties of SP cells.

**Figure 5:**
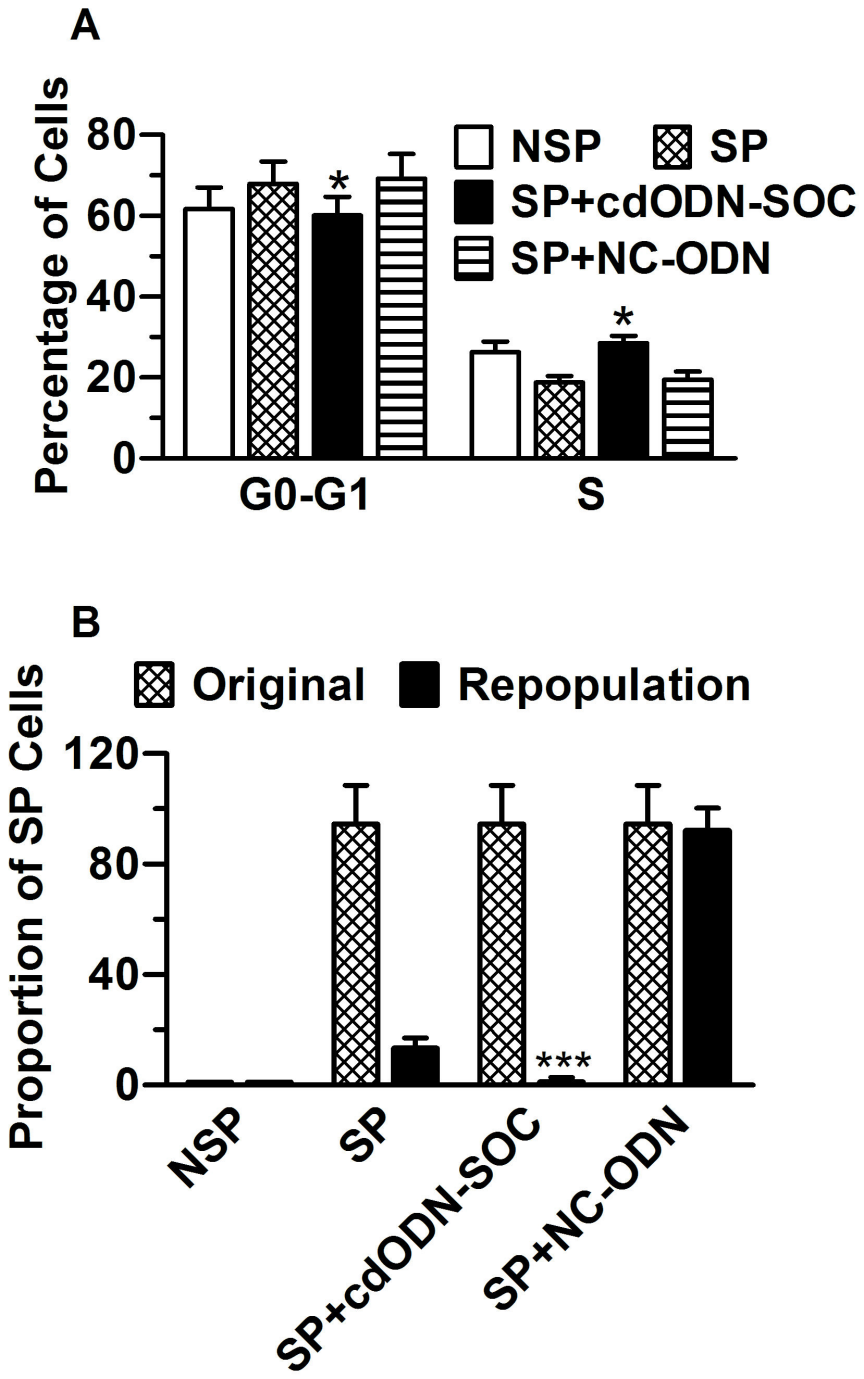
Cell cycle characteristics and differentiation ability of PANC-1 SP and NSP cells. (**A**) Percentage of PANC-1 SP and NSP cells in G0-G1 and S cell cycles. Note that cdODN-SOC minimized the difference between SP and NSP. *P<0.05 vs SP alone; n=4. (B) Freshly sorted PANC-1 SP and NSP cells were immediately reanalyzed to ensure the purity, and were cultured for 1 week before re-staining with Hoechst33342 dye and resorted. Note that the SP cells repopulated both SP and significant NSP proportions from the original sort, whereas the NSP generated only NSP cells, and cdODN-SOC eliminated the ability of SP cells to repopulate SP cells. ***P<0.001 *vs* SP alone; n=4.

We isolated SP and NSP cells from the three h-PCCLs and plated an equal density of cells from both populations in triplicate in 96-well plates. Growth of the cells was measured using the MTT method. The data showed that the proliferation rate of SP cells was not significantly different from NSP cells (*P*>0.05). Yet, the freshly sorted SP cells tended to have a smaller proportion of cells in the S phase, but a higher percentage of cells in the G1 phase, compared to the NSP cells. Transfection with cdODN-SOC minimized the differences (Figure **5A**).

We then turned to assess the effects of cdODN-SOC on the repopulation ability of SP cells to generate both SP and NSP cells *in vitro*. To this end, we cultured the sorted SP and NSP cells from the three h-PCCLs separately under the same culture condition for 10 days, followed by re-staining with Hoechst33342 dye. Both fractions were viable in culture, but in the absence of cdODN-SOC the PANC-1 SP cells generated both SP and NSP with a fraction size comparable with the original population: the SP proportion was significantly decreased from 94.5±1.4 to 13.4±3.6%, whereas the NSP cells produced mainly NSP cells, generating only ~1% SP cells (Figure **5B**). When the cells were treated with cdODN-SOC, virtually no SP proportion was generated by SP cells. NC–ODN did not affect the SP-repopulation ability of SP cells. Similar results were observed in SW1990 and BxPc-3 cells.

**Figure 6:**
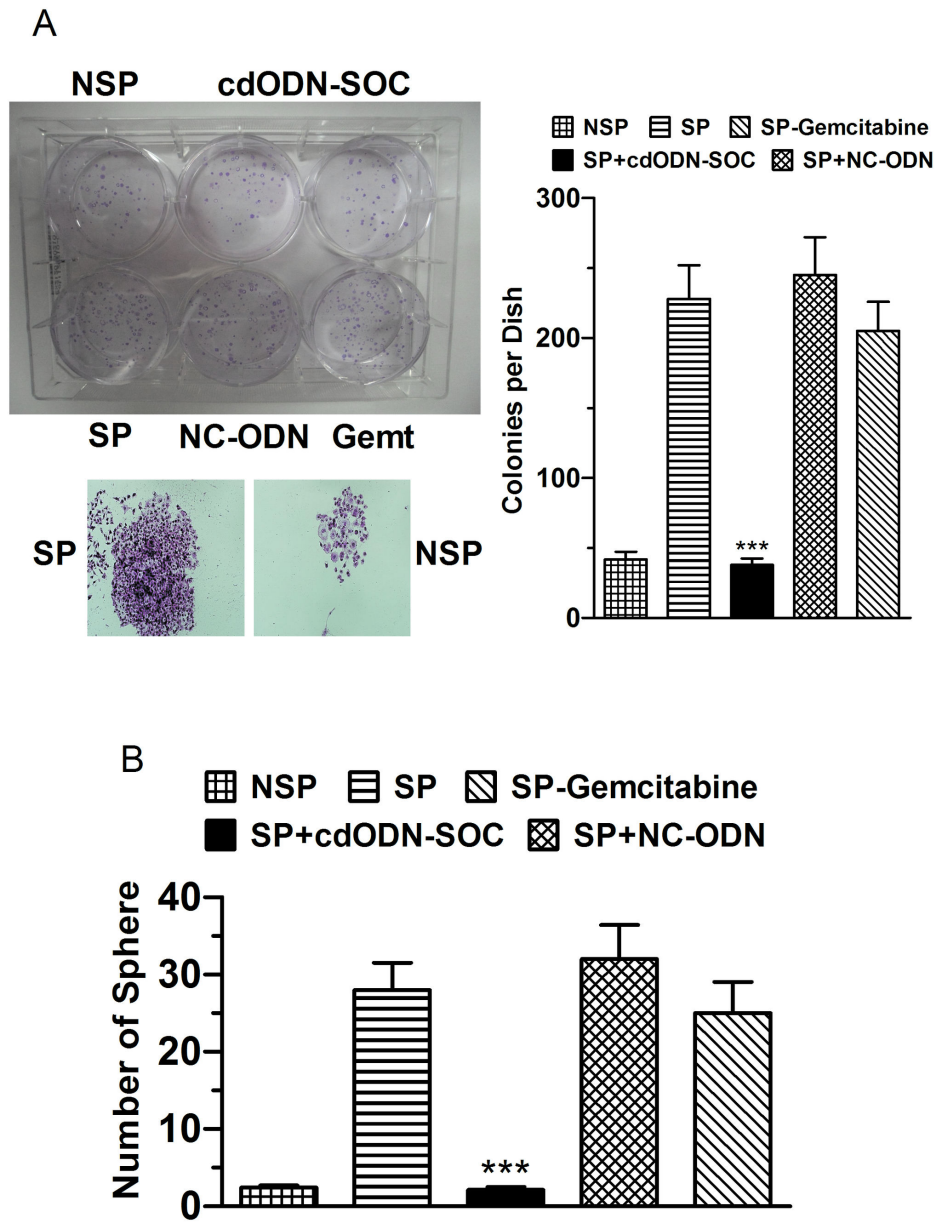
Suppression of the clonogenic ability of CSCs in h-PCCLs by cdODN-SOC. (**A**) Upper left: representative examples of soft agar assays for clonogenic ability of PANC-1 CSCs under varying conditions and lower left: the amplified view of colonies. Right panel: mean data showing the effects of cdODN-SOC on the number of colonies. SP cells were more clonogenic than NSP cells and cdODN-SOC suppressed the clonogenic ability. ***p<0.001 vs SP alone. (B) Serial sphere formation assays for clonogenic ability of CSCs, showing the suppressive effects of cdODN-SOC on spherical formation. ***p<0.001 vs SP alone. Quantitatively the same results were obtained from SW1990 and BxPc-3 CSCs (data not shown).

The effects of cdODN-SOC on the clonogenic ability of SP and NSP cells were examined when seeded as single cells. The clonogenic efficiency of SP cells was significantly higher than NSP cells in soft agar assays (Figure **6A**). Moreover, most SP cells divided into colonies of more than 50 cells in soft agar after three weeks. There was no apparent difference between NSP cells and SP cells. The self-renewal ability of SP cells was also examined in serial soft agar assays. There was an increase in clonogenic efficiency from primary to secondary colonies. Moreover, secondary colonies were similar in size to the primary colonies, indicating that SP cells can maintain and expand themselves in serial soft agar assays. Notably, cdODN-SOC considerably suppressed the clonogenic ability of SP cells, whereas NC-cdODN failed to affect the clonogenesis.

We continued to evaluate the effects of cdODN-SOC on the ability of SP and NSP cells to generate spherical clones and self-renewal by sphere formation assays. SP cells formed typical floating spheres with an efficiency of 6.2±0.7%, whereas the NSP cells mostly showed adherent growth pattern with much lower sphere-forming capacity (0.7±0.2%). Transfection of cdODN-SOC reduced the efficiency to 1.3±0.3% in SP cells and to 0.5±0.2% in NSP cells. When the primary SP cell-derived spheres were dissociated and passaged, they readily formed secondary spheres, and the secondary spheres occurred slightly more quickly and frequently than the primary spheres (Figure **6B**), suggesting that SP cells have self-renewal ability. CdODN–SOC nearly abolished the formation of secondary spheres, but NC–ODN did not alter sphere formation at all.

**Figure 7:**
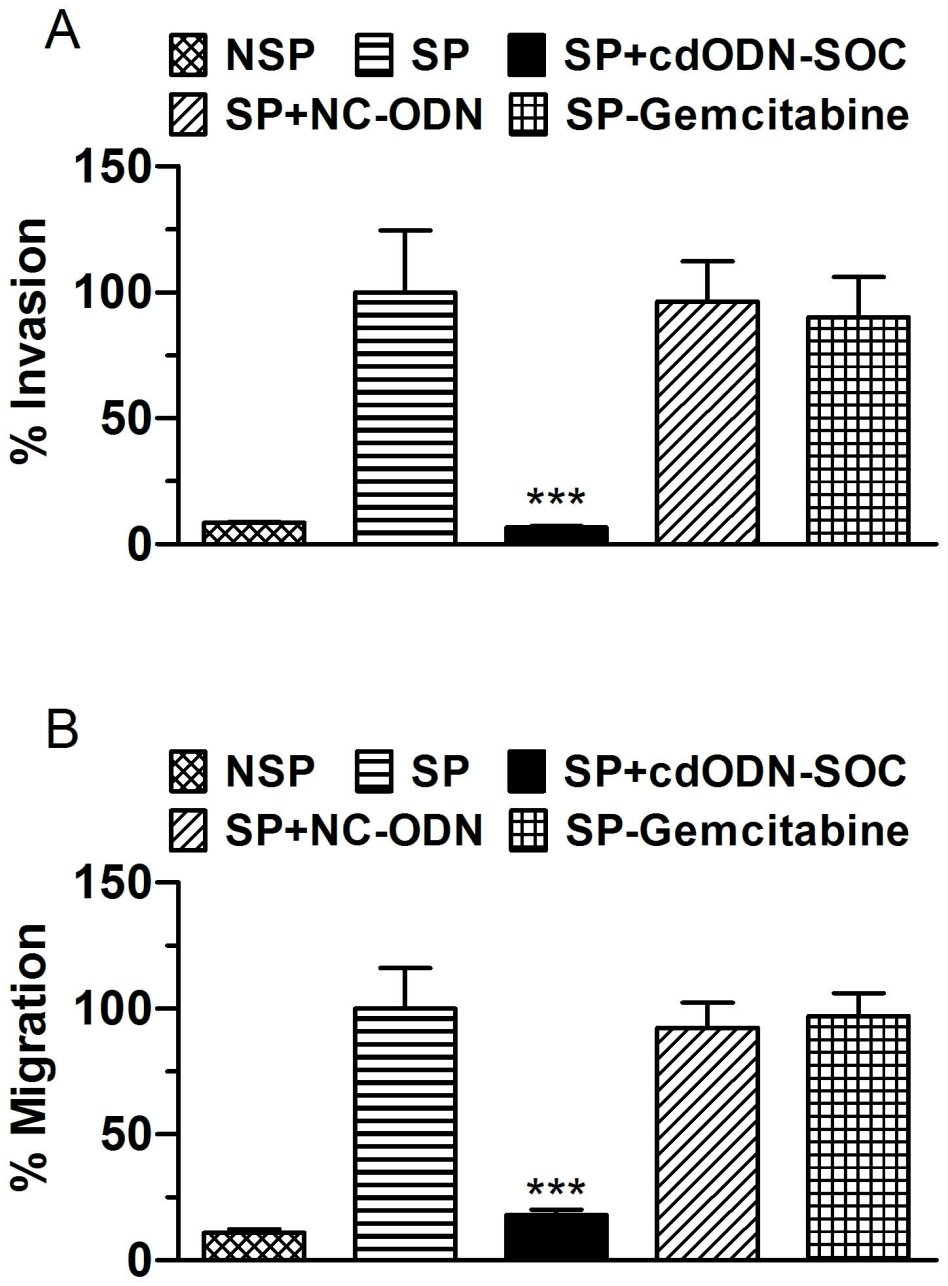
Inhibition of SP cell invasion and migration by cdODN-SOC. (**A**) Invasion assay. PANC-1 CSCs were plated onto the Matrigel-coated membrane in the top chamber of the transwell and transfected or treated with gemcitabine for 48 h. All cells were mock-treated with lipofectamine 2000. Cells invaded to the lower chambered were fixed with methanol, stained with crystal violet and counted. ***p<0.001 vs SP alone; n=6. (B) Migration assay. PANC-1 CSCs were plated in the top chamber of the transwell and transfected or treated with gemcitabine for 36 h. All cells were mock-treated with lipofectamine 2000. Cells migrated to the lower chambered were fixed with methanol, stained with crystal violet and counted. ***p<0.001 vs SP alone; n=5. Quantitatively the same results were obtained from SW1990 and BxPc-3 CSCs (data not shown).

Figure **7C** shows that SP cells of the three h-PCCLs all had substantially higher percentages of invading cells and migrating cells than the NSP cells, which were suppressed by cdODN-SOC.

#### CdODN–SOC eliminates the drug resistance of human pancreatic CSCs

**Figure 8:**
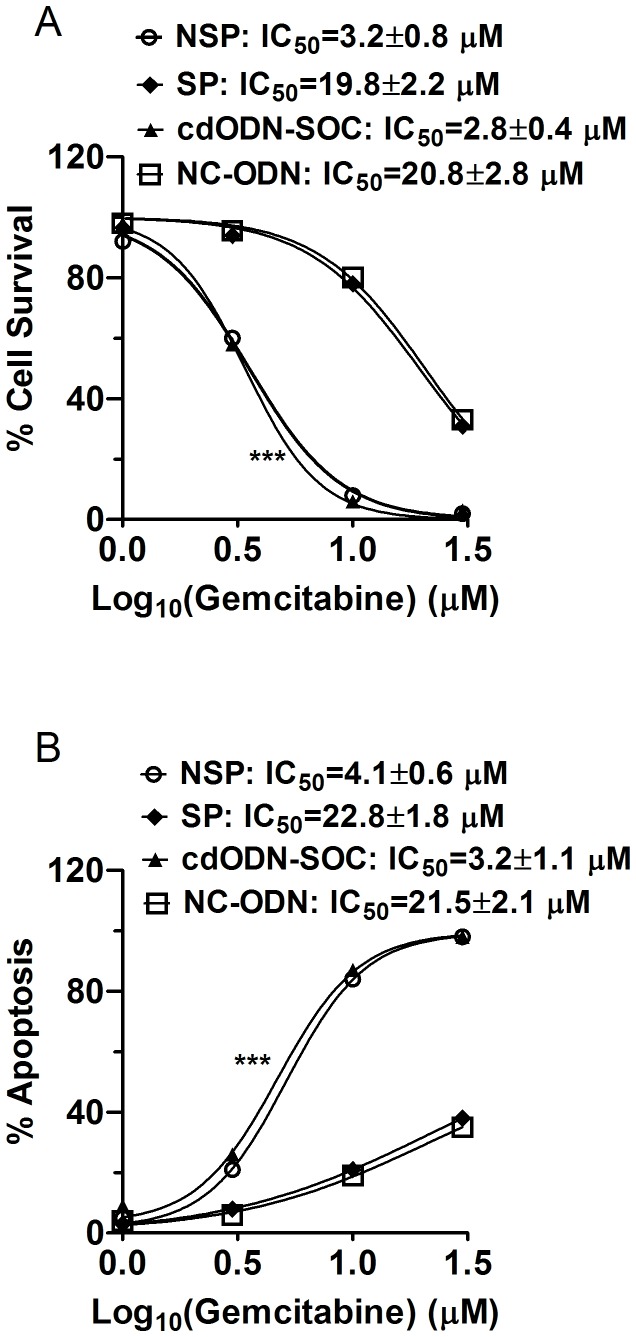
Enhanced drug resistance of SP cells and suppression by cdODN-SOC. (**A**) Percent cell survival measured by MTT assay in PANC-1 CSCs. All cells were all treated with varying concentrations of gemcitabine and with lipofectamine 2000. Symbols are experimental data and the lines represent best fits to Hill equation. ***p<0.001 vs SP alone for comparison of IC50 values; n=4. (B) Percent apoptotic cells measured by ELISA to quantify DNA fragmentation in PANC-1 CSCs. All cells were all treated with varying concentrations of gemcitabine and with lipofectamine 2000. Symbols are experimental data and the lines represent best fits to Hill equation. ***p<0.001 vs SP alone for comparison of IC50 values; n=4. Quantitatively the same results were obtained from SW1990 and BxPc-3 CSCs (data not shown).

Sorted SP and NSP cells from the three h-PCCLs were treated with varying concentrations of gemcitabine (1, 3, 10, and 30 µM), and. The concentration for half maximal inhibition (IC_50_) of cell survival was calculated based on the dose–response curve fitted by Hill equation. The SP cells had a higher IC_50_ than the NSP cells (19.8±2.2 µM vs. 3.2±0.8 µM; n=4, P=0.0044) (Figure **8A**). To further evaluate the difference between the SP and NSP cells with regards to the induction of apoptosis, quantification of DNA fragmentation was performed using ELISA methods following gemcitabine treatments to determine gemcitabine-induced apoptosis. Consistent with cell survival data, SP cells displayed greater resistance to apoptosis induction than NSP cells (Figure **8B**), indicating that the SP cells were more resistant to gemcitabine.

Notably, in the presence of cdODN-SOC, the SP cells entirely lost the resistance to gemcitabine: the IC_50_ values were reduced by one order of magnitude. For example, for PANC-1 cells the IC_50_ for decreased cell survival was reduced from 19.8±2.2 µM to 2.8±0.8 µM and the IC_50_ for apoptosis was reduced from 22.4±1.8 µM to 3.2±1.1 µM (Figure 8A & 8B).

### CdODN–SOC suppresses tumorigenicity of human pancreatic CSCs

**Figure 9:**
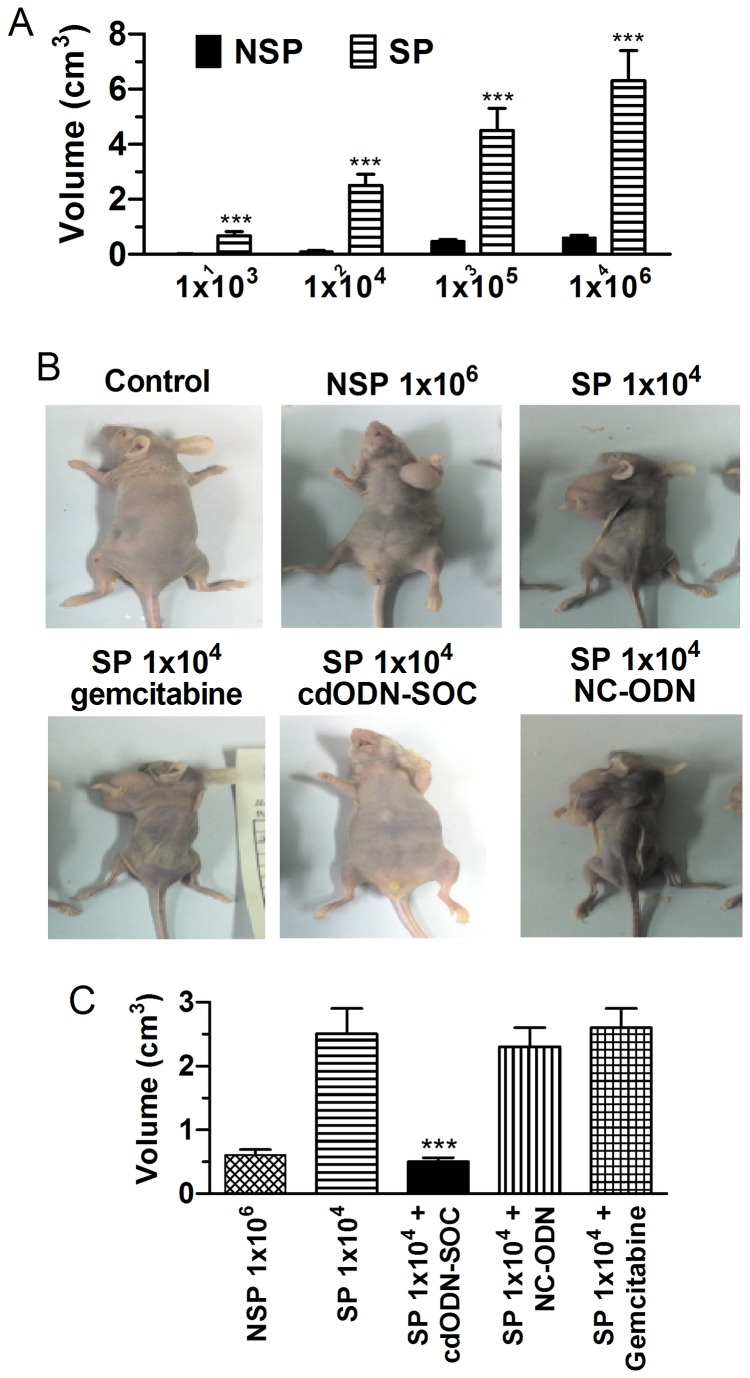
Enhanced *in vivo* tumorigenicity of SP cells and the anti-tumor effects of cdODN-SOC in BALB/C nude mice. PANC-1 SP and NSP cells were orthotopically inoculated s.c. to the left dorsal flank of mice. On 50 days after inoculation, the mice were sacrificed to detect tumor formation. (**A**) The SP cells regenerated larger tumors than corresponding NSP cells at every cell dose. ***p<0.001 SP vs NSP; n=6. (B) Representative images of tumors in nude mice showing the difference in the tumorigenic potentials between SP and NSP and the anti-tumor effects of cdODN-SOC, as well as the drug resistance of SP-induced tumor to gemcitabine. For the PANC-1 cell line, as few as 1×104 SP cells formed tumors, while 1×106 NSP cells were required to initiate a tumor. (C) Mean data of tumor volume under different conditions. Note the ability of cdODN-SOC to reduce the tumor volume. ***p<0.001 vs SP; n=6.

The tumorigenicity of SP and NSP cells from all three h-PCCLs was compared in nude mice. For PANC-1 inoculation, 1×10^6^ NSP cells gave rise to new tumors in only 1/4 mice tested; however, SP cells formed a tumor in all 4 mice studied when only 1×10^4^ cells were inoculated (Figure **9A**). Furthermore, liver metastasis was detected in 3/5 mice inoculated with 1×10^5^ SP cells. Qualitatively the same results were observed in other two h-PCCLs (Figure **9B**). Additionally, the tumor volume was greater in SP than in NSP tumors (Figure **9C**). The tumorigenic potential of SP cells was substantially suppressed by cdODN-SOC. First, the SP cells pretreated with cdODN-SOC lost the ability to grow tumors in nude mice (Figure **9A**) even at an inoculation density of 1×10^6^. Second, intratumoral injection of cdODN-SOC markedly retarded the tumor growth, as indicated by the significantly reduced tumor volume (Figure **9C**).

Treatment of mice with gemcitabine affected growth of only 1 out of 5 xenograft tumors using 50% reduction of SP tumor volume as positive response. This effect resulted in an average volume reduction of 63% in comparison with an average tumor expansion in the vehicle-treated control group of 190% (p=0.022; Figure **9D, E**). The tumor SP proportion was larger in mice treated with gemcitabine than in the corresponding controls (median 6.6% versus 2.7%, respectively; n=7; p=0.028; Figure **9B-C**). SP enrichment was even higher when only considering the tumors that responded to gemcitabine with tumor shrinkage (of at least 50%). Whereas these results clearly indicated the drug resistance of the tumor cells to gemcitabine, all tumors responded to cdODN-SOC, suggesting that no resistance had been developed against cdODN-SOC.

## Discussion

Here we characterized three human pancreatic cancer cell lines (h-PCCLs) PANC-1, SW1990, and BxPc-3 for the presence and properties of cancer stem cells (CSCs) and assessed the effects of a artificially designed decoy molecule that was designed to target multiple transcription factors on the CSCs. The main findings in the present study include: (1) CSCs are enriched in the side proportion (SP) cells contained in the h-PCCLs, though to different extents in different PCCLs, and they possess aggressive growth, invasion, migration and drug-resistance properties and (2) a single decoy simultaneously targeting Sox2, Oct4 and c-Myc (cdODN-SOC) efficiently suppressed the CSC properties, leading to elimination of CSCs phenotypes in h-PCCLs or loss of stemness of SP cells in h-PCCLs. The findings indicate that targeting the key genes conferring the CSC identity is able to convert the CSC to non-SP (NSP) cells, and thus may be considered a new and alternative approach for cancer therapy. Specifically, the present study establishes the combination of Sox2/Oct4/c-Myc targeting as a potential anticancer agent worthy of further studies in preclinical settings.

The presence of SP fraction in PANC-1, SW1990, and BxPc-3 cells and the CSC-like properties of these SP cells have been documented by other groups [7–14,23]. We provided both in vitro and *in vivo* evidence for the ability of the SP cells from h-PCCLs to regenerate a population of cells comprised of both SP and NSP, indicating the self-renewal ability to produce heterologous descendent cells by asymmetric division. When inoculated into nude mice, the SP cells were found to be more tumorigenic than NSP cells and to be able to cause liver metastases. These observations are also consistent with the findings in previous studies, underscoring the importance of CSCs in the development, as well as therapy, of PDAC.

One of the major challenges in the treatment of PDAC is the frequent failure of chemotherapy. The lack of success with existing strategies underscores the importance of continued research efforts, in the hope of developing novel therapeutic strategies for the treatment of patients diagnosed with PDAC. According to the CSC theory [3], conventional chemotherapies and radiation therapy kill differentiated or differentiating cells, and these cells form the bulk of the tumor, but cannot generate new cells. Tumor relapse may occur because CSCs remain non-attacked by drugs, suggesting the removal of CSCs is crucial for effective cancer therapy. Unlike most cells within the tumor, CSCs, including pancreatic CSCs, are resistant to chemotherapy and may contribute to tumor metastasis and tumor recurrence after treatment. Therefore, drugs that selectively target CSCs offer a greater promise for cancer therapy and/or prevention. Many pharmaceutical companies are investing heavily in developing new therapeutics to target cancer stem cells, and more efficient inhibitors of several developmental signaling pathways are currently being developed or in the early phases of testing [24]. A key issue needed to be worked out before embarking on clinical trials to target pancreatic cancer stem cells will be determining the best way to measure the efficacy of these new therapies. Traditionally, the effectiveness of cancer agents is measured by tumor shrinkage. Tumor response is usually defined as tumor shrinkage by at least 50%. If cancer stem cells are resistant to therapy and make up a very small percentage of cells within the tumor, the effect of therapeutics may reflect the effect on the differentiated, nontumorigenic cancer cells, rather than cancer stem cells. For clinical trials testing pancreatic cancer stem cell therapeutics, new measures of efficacy will need to be devised.

It is known that transcription factors make up 6% of the human genome, ranking second because of their abundance, and have recently been considered a new class of candidate targets for drug discovery [25]. The decoy technology using these transcription factors as molecular targets has been emerged as a vivid strategy for gene therapy of a broad range of human diseases [26,27]. Moreover, Gao et al. in 2006 [18] developed the ‘one-drug, multiple-target’ concept and established the anticancer efficacy of the complex decoy ODN (cdODN) that targets oncoproteins NF-κB, E2F and Stat3. In the present study, we extended this pioneer work to containing *cis*-elements for pluripotency maintaining factors Sox2, Oct4 and c-Myc to target CSC-like phenotype. Nanog, Sox2, and Oct4 are transcription factors all essential to maintaining the pluripotent embryonic stem cell phenotype [28]. Sox2 binds specifically to a 7-bp sequence termed the HMG site and this site is often found adjacent to a binding site for Oct-3/4 (termed the POU site). Indeed, Oct-4 and Sox2 bind to the Nanog promoter in living mouse and human ESCs [28]. Nanog, Oct-4 and Sox2 co-occupy and regulate their own promoters together with other developmental genes with diverse functions and collaborate to form an extensive regulatory circuitry including autoregulatory and feed-forward loops [28,29]. Recent studies revealed that through a cooperative interaction, Sox2 and Oct4 have previously been described to drive pluripotent-specific expression of a number of genes including Nanog. On the other hand, it has been shown that the endothelial cells expressing Nanog also express other stemness genes including Sox2, Oct4, and c-Myc, which are not normally expressed or are expressed at very low levels in these cells [30]. Sox2 also regulates expression of c-Myc, Wnt1, Wnt2, and Notch1 in xenografted NOD/SCID mice [31]. Oct4 has been shown to mediate chemoresistance through a potential Oct4-Akt-ABCG2 pathway [21,22,32]. C-Myc is essential for keeping CSC-like phenotype in human hepatocellular carcinoma (HCC) [33]. Clearly, there is a complex network constantly operating in SP cells to maintain the pluripotent embryonic stem cell phenotype and to confer the CSC-like properties such as aggressive invasion, migration and multidrug resistance. Hence, by targeting Sox2, Oct4 and c-Myc, a wide spectrum of signaling molecules crucial to tumorigenesis are also deemed to be affected directly or indirectly.

Recent evidence suggests a shared genomic fingerprint between embryonic stem cells, cancer cells, and cancer stem cells. Activation targets of Nanog, Oct-4, Sox2 and c-Myc are more frequently overexpressed in many tumors. The advantages of the cdODN approach are likely to be ascribed to simultaneous interference of expression of multiple genes controlled by the target transcription factors: in our case, sox2, Oct4, and c-Myc which critically determine the CSC identity and properties. According to Gao et al. [18], the cdODN one-drug, multiple-target strategy mimics the well known drug cocktail therapy, but it is advantageous over the latter in that it is devoid of the weaknesses of the drug cocktail therapy, involving complicated treatment regimens, undesirable drug–drug interactions, and increased side effects. Additionally, the cdODN strategy offers resourceful combinations of varying *cis* elements for concomitantly targeting multiple transcription factors that control expression of many distinct but interrelated genes. The cdODN-SOC tested in this study is potentially applicable to a wide spectrum of cancers, as the target transcription factors/pluripotency maintaining factors Sox2, Oct4 and c-Myc are not cell/tissue specific; they are overexpressed in SP cells of various origins [10,15,34–40]. It is there plausible that our cdODN-SOC may also be applied to cancers of various origins other than PDAC. Yet this notion definitely requires future studies for verification.

## Supporting Information

Figure S1 **Differential gene expression between SW1990 SP and NSP cells.** (**A**) Expression of CD133, ALDH1, ABCG2, Sox2, Oct4, Nanog, c-Myc, and Notch1 at the mRNA level, determined by real-time RT-PCR. The values were obtained by first normalized to GAPDH for internal control and then presented as a ratio of SP over NSP. **p<0.01 SP vs NSP; ***p<0.001 SP vs NSP; n=4. (B) Expression of CD133, ALDH1, ABCG2, Sox2, Oct4, Nanog, c-Myc, and Notch1 at the protein level, determined by Western blot analysis. The values were obtained by first normalized to GAPDH for internal control and then presented as a ratio of SP over NSP. CD133: 120 kDa; ALDH1: 55 kDa; ABCG2: 72 kDa; Sox2: 40 kDa; Oct4: 45 kDa; Nanog: 35 kDa; c-Myc: 62 kDa; Notch1: 300 kDa. ***p<0.001 SP *vs* NSP; n=4.
(TIF)

Figure S2 **Differential gene expression between BxPc-3 SP and NSP cells.** (A) Expression of CD133, ALDH1, ABCG2, Sox2, Oct4, Nanog, c-Myc, and Notch1 at the mRNA level, determined by real-time RT-PCR. The values were obtained by first normalized to GAPDH for internal control and then presented as a ratio of SP over NSP. ***p<0.001 SP vs NSP; n=4. (B) Expression of CD133, ALDH1, ABCG2, Sox2, Oct4, Nanog, c-Myc, and Notch1 at the protein level, determined by Western blot analysis. The values were obtained by first normalized to GAPDH for internal control and then presented as a ratio of SP over NSP. CD133: 120 kDa; ALDH1: 55 kDa; ABCG2: 72 kDa; Sox2: 40 kDa; Oct4: 45 kDa; Nanog: 35 kDa; c-Myc: 62 kDa; Notch1: 300 kDa. ***p<0.001 SP vs NSP; n=4.
(TIF)
