# Expression of Concern: Concomitant Targeting of Multiple Key Transcription Factors Effectively Disrupts Cancer Stem Cells Enriched in Side Population of Human Pancreatic Cancer Cells

**DOI:** 10.1371/journal.pone.0174194

**Published:** 2017-03-14

**Authors:** The PLOS ONE Editors

Concerns have been raised regarding the in vivo experiments reported in this study and specifically regarding the procedures involved in the experiments testing tumorigenicity in mice:

1. The Methods section describing the tumorigenicity assay states that “The mice were sacrificed at day 50 or when the tumors grow to a maximum of 1,000 mm^3^”. However, the charts in Figure 9A,C show tumor volumes in several experimental groups exceeding this limit; in one group, tumor sizes >6cm^3^ were reported. The representative photographs in Figure 9B also show mice with very large tumor burdens. The tumor sizes reported in Figure 9 exceed the limits that are generally deemed acceptable according to international animal research ethics guidelines, and suggest that the humane endpoint (1000 mm^3^) described in the Methods section was not applied.

2. In the Methods it is stated that the cells were injected to the dorsal flank of the mice but the location is inconsistent with where the tumors appear in Figure 9B.

3. There are concerns about whether the tumor volumes were calculated correctly.

The PLOS ONE Editors have contacted the authors to request information on the procedures employed for the handling of the animals as well as tumor dimension measurements, we have not received a response to our requests. We have also contacted Xinjiang Medical University and Sun Yat-sen University in relation to our concerns over the animal experiments reported in the article.

In light of the concerns raised and the information available, we have concerns that the work may not have met PLOS ONE's ethical standards and our requirements for reporting and methodological rigor. We are thus issuing this Expression of Concern to alert readers of the concerns identified about this article.
