# Supplementary figures and images for: Concomitant Targeting of Multiple Key Transcription Factors Effectively Disrupts Cancer Stem Cells Enriched in Side Population of Human Pancreatic Cancer Cells
Source: PLoS One. 2013 Sep 11;8(9):e73942. doi: 10.1371/journal.pone.0073942 (PMC3770686; doi:10.1371/journal.pone.0073942)

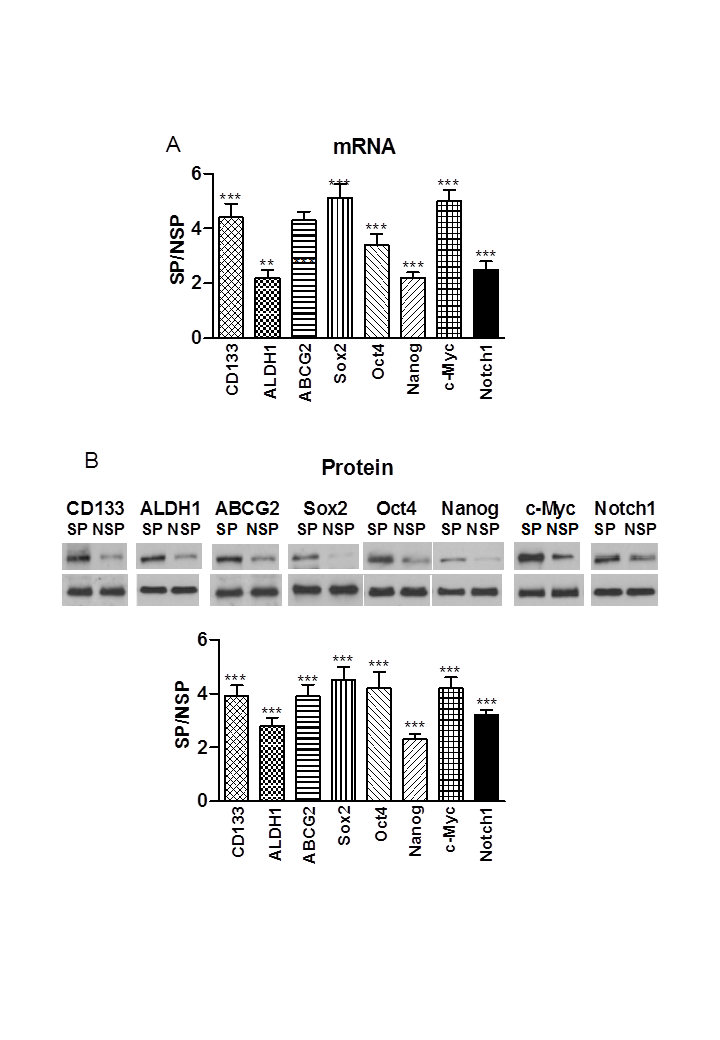

Supplement: Figure S1 — Differential gene expression between SW1990 SP and NSP cells. (A) Expression of CD133, ALDH1, ABCG2, Sox2, Oct4, Nanog, c-Myc, and Notch1 at the mRNA level, determined by real-time RT-PCR. The values were obtained by first normalized to GAPDH for internal control and then presented as a ratio of SP over NSP. **p<0.01 SP vs NSP; ***p<0.001 SP vs NSP; n=4. (B) Expression of CD133, ALDH1, ABCG2, Sox2, Oct4, Nanog, c-Myc, and Notch1 at the protein level, determined by Western blot analysis. The values were obtained by first normalized to GAPDH for internal control and then presented as a ratio of SP over NSP. CD133: 120 kDa; ALDH1: 55 kDa; ABCG2: 72 kDa; Sox2: 40 kDa; Oct4: 45 kDa; Nanog: 35 kDa; c-Myc: 62 kDa; Notch1: 300 kDa. ***p<0.001 SP vs NSP; n=4. (TIF) [file pone.0073942.s001.tif]

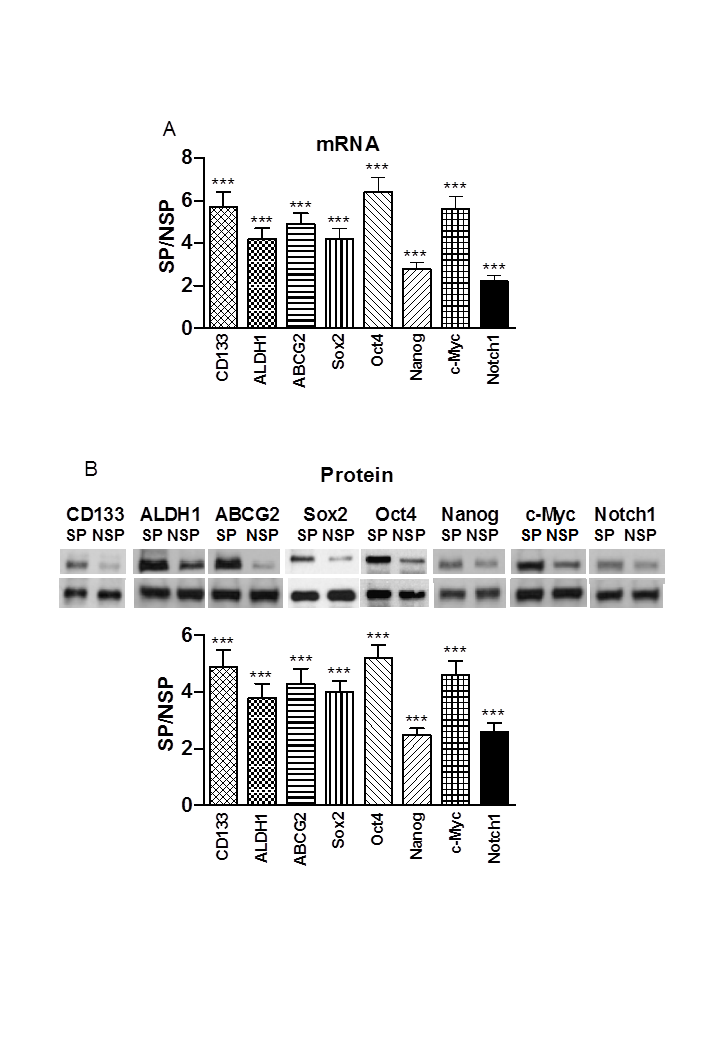

Supplement: Figure S2 — Differential gene expression between BxPc-3 SP and NSP cells. (A) Expression of CD133, ALDH1, ABCG2, Sox2, Oct4, Nanog, c-Myc, and Notch1 at the mRNA level, determined by real-time RT-PCR. The values were obtained by first normalized to GAPDH for internal control and then presented as a ratio of SP over NSP. ***p<0.001 SP vs NSP; n=4. (B) Expression of CD133, ALDH1, ABCG2, Sox2, Oct4, Nanog, c-Myc, and Notch1 at the protein level, determined by Western blot analysis. The values were obtained by first normalized to GAPDH for internal control and then presented as a ratio of SP over NSP. CD133: 120 kDa; ALDH1: 55 kDa; ABCG2: 72 kDa; Sox2: 40 kDa; Oct4: 45 kDa; Nanog: 35 kDa; c-Myc: 62 kDa; Notch1: 300 kDa. ***p<0.001 SP vs NSP; n=4. (TIF) [file pone.0073942.s002.tif]
